# Efficacy and safety of eptinezumab in patients with chronic migraine and medication-overuse headache: a randomized, double-blind, placebo-controlled study

**DOI:** 10.1186/s12883-023-03477-z

**Published:** 2023-12-15

**Authors:** Shengyuan Yu, Jiying Zhou, Guogang Luo, Zheman Xiao, Anders Ettrup, Gary Jansson, Ioana Florea, Kristina Ranc, Patricia Pozo-Rosich

**Affiliations:** 1 Department of Neurology, Chinese PLA General Hospital, Beijing, China; 2 Department of Neurology, The First Affiliated Hospital of Chongqing Medical University, Chongqing, China; 3 Neurology Department, The First Affiliated Hospital of Xi’an Jiaotong University, Xi’an, China; 4 Department of Neurology, Renmin Hospital of Wuhan University, Wuhan, China; 5 H. Lundbeck A/S, Copenhagen, Denmark; 6 Neurology Department, Headache Unit, Vall d’Hebron University Hospital, Barcelona, Spain; 7 Headache and Neurological Pain Research Group, Vall d’Hebron Research Institute, Department de Medicina, Universitat Autonoma de Barcelona, Barcelona, Spain

**Keywords:** Chronic migraine, Medication-overuse headache, Anti-CGRP, Eptinezumab, Preventive migraine treatment

## Abstract

**Background:**

For some people with migraine, despite taking greater amounts of acute headache medication (AHM), they develop an increase in monthly headache days. This cycle of increasing headache days, and in turn AHM use, can lead to a secondary headache disorder called medication-overuse headache (MOH). Preventive medications can prevent migraine from occurring and reduce reliance on AHMs, thereby preventing the cycle of MOH. This study was performed to evaluate the efficacy and safety of eptinezumab to prevent migraine/headache in a mainly Asian patient population with a dual diagnosis of chronic migraine and MOH.

**Methods:**

SUNLIGHT was a phase 3, multicenter, double-blind, parallel-group, placebo-controlled trial. Patients aged 18−75 years with ≥ 8 migraine days/month and a diagnosis of MOH were randomly allocated (1:1) to one of two treatment groups: eptinezumab 100 mg or placebo. Monthly migraine days (MMDs) were captured using a daily electronic diary; the change from baseline in the number of MMDs over Weeks 1−12 was the primary efficacy endpoint.

**Results:**

Patients were randomized to eptinezumab 100 mg (*n* = 93) or placebo (*n* = 100). Over Weeks 1−12, eptinezumab reduced mean MMDs more than placebo (difference between treatments was -1.2; *p* = 0.1484). Differences between treatment groups with p-values below 0.05 favoring eptinezumab were observed in 3 out of the 6 key secondary endpoints.

**Conclusion:**

All endpoints numerically favored eptinezumab treatment when compared to placebo; however, this study did not meet its primary endpoint and is therefore negative. No new safety signals were identified in this study, like previous reports that confirmed the safety and tolerability of eptinezumab treatment.

**Trial registration:**

ClinicalTrials.gov identifier: NCT04772742 (26/02/2021).

**Supplementary Information:**

The online version contains supplementary material available at 10.1186/s12883-023-03477-z.

## Background

Migraine, a common and disabling neurologic disorder [1, 2], is one of the leading causes of global disability [3, 4]. Migraine, especially chronic migraine (CM; when a patient has headache [migraine-like or tension-type–like] on ≥ 15 days/month for ≥ 3 consecutive months, which on ≥ 8 days/month has the features of migraine headache) [5], can negatively impact all aspects of daily life and is associated with many comorbidities, including: major depression, anxiety, cardiac disorders, respiratory disorders, non-headache pain, and others [6, 7]. Disease progression entails that some patients will develop an increase in monthly headache days (MHDs) despite taking greater amounts of acute headache medication (AHM). This cycle of increasing headache days and in turn more AHM use can lead to a secondary headache disorder called medication-overuse headache (MOH) [5, 8].

Globally, MOH is estimated to occur in up to 70% of individuals with CM and is considered a risk factor for migraine chronification, where episodic migraine becomes CM [9–11]. In China, the prevalence of MOH within the migraine patient population is high, with hospital-based studies from 2013–2015 reporting that approximately 40–71% of CM patients also had MOH [12, 13]. In Europe, patients with migraine and MOH generally constitute the most burdensome population, linked to approximately 86% of all healthcare costs generated by patients with headache disorders [14]. Moreover, patients with a dual diagnosis of CM and MOH often face the largest amount of burden and impact on quality of life [15].

Preventive migraine medication can prevent migraine attacks from occurring, reducing reliance on ineffective or poorly tolerated AHM and thereby breaking the cycle of MOH. However, there is a need for preventive medications that are more effective and better tolerated than the current standard of care [16]. The calcitonin gene-related peptide (CGRP) antagonist eptinezumab [17] is a peptide-binding IgG1 antibody that inhibits migraine onset; [18]; it has proven efficacy in adults with episodic migraine [19], with CM [20], and with 2–4 previous preventive migraine treatment failures [21]. Moreover, in subgroup analyses of patients with both CM and MOH, eptinezumab demonstrated efficacy in reducing monthly migraine days (MMDs), AHM use, and the impact of migraine as measured by patient-reported outcomes [8, 22, 23]. Key pharmacologic attributes of eptinezumab include high selectivity and affinity for CGRP, intravenous (IV) formulation, and short time to maximum plasma concentration (around 30 min) [24, 25]. When compared to oral acute treatment(s), the IV route of administration avoids first pass metabolism in the liver and kidneys, allowing for a faster onset, which may be of high importance in this patient population. The objective of SUNLIGHT was to evaluate the efficacy and safety of eptinezumab to prevent migraine and headache in a primarily Asian patient population with the dual diagnosis of CM and MOH. Here, we report the primary results of the SUNLIGHT study and discuss factors potentially contributing to the study results.

## Methods

### Study design

SUNLIGHT was a randomized, double-blind, parallel-group, placebo-controlled phase 3 clinical trial that enrolled patients with a dual diagnosis of migraine and MOH for the purpose of evaluating the efficacy of eptinezumab within this specific demographic. This multicenter study was conducted in accordance with Good Clinical Practice standards as defined by the International Conference on Harmonisation and all applicable federal and local regulations. Each study site’s local review board or alternatively a central institutional review board/ethics committee approved all study documents. Patients were recruited from specialist settings in Mainland China, Republic of Korea, Taiwan, Spain, and Georgia. All patients provided written informed consent prior to their participation in the study. To view this registered study, see ClinicalTrials.gov under the following identifier: NCT04772742 (26/02/2021).

In this 36-week study, patients were followed through a screening period (28–30 days), a placebo-controlled period measuring efficacy (12 weeks), and an open-label period measuring safety and tolerability (12 weeks). For patients entering the open-label period, safety was followed for 20 weeks (12 weeks during the open-label period and 8 weeks during the safety follow-up period). These 20 weeks of safety data were analyzed and presented together. For patients not entering the open-label period, 20 weeks of safety follow-up data counted from when they received the first study drug infusion. The safety data collected up to Week 12 are thus included in the tabulations of the placebo-controlled period safety data, whereas the data collected at the safety follow-up visit for these patients are reported separately in data listings (Supplemental Figure 1). Patients were randomized to receive either eptinezumab 100 mg or placebo by IV infusion at the baseline visit. Eptinezumab (100 mg) was dispensed as 1 vial of 100 mg/mL concentrate for solution for infusion; 1 ml of 100 mg/ml concentrate for solution for infusion was added to 100 mL of 0.9% normal saline. Placebo was dispensed as 100 mL of 0.9% normal saline. The pharmacist or designee who received, stored, prepared, and dispensed eptinezumab and placebo IV infusions was unblinded and not involved in clinical study activities for which blinding was needed. The blinded investigator or designee intravenously administered study drug or placebo, which took approximately 30 min (± 15 min). At the primary outcome visit (Week 12), all patients received an IV infusion with eptinezumab 100 mg.

Patients were assigned an electronic headache diary, called an eDiary, at the screening visit and were required to complete daily entries from the screening visit to the primary outcome visit (Week 12) or until the withdrawal visit. Patients used the eDiary in their local language to record information regarding any experienced headaches, such as start time, stop time, headache severity, additional symptoms, and acute headache/migraine medication use. The yes/no responses to headache items and the severity rankings (mild, moderate, or severe) helped investigators track any effects of treatment. Migraine was ranked as either moderate or severe. Information collected from the eDiary was used to derive headache/migraine study endpoints.

### Patient population

Adults 18–75 years old (inclusive) with migraine onset at 50 years old or younger were eligible for participation if their migraine diagnosis met the criteria established in the International Classification of Headache Disorders, 3^rd^ edition (ICHD-3) guidelines: a history of migraine onset ≥ 12 months prior to the screening visit, ≥ 8 migraine days per month for the 3 months prior to the screening visit, and a diagnosis of MOH as defined by ICHD-3 guidelines (i.e., the patient had headache on ≥ 15 days/month for the past 3 months prior to the screening visit and had regular overuse of one or more drugs that can be taken for acute and/or symptomatic treatment of headache, for > 3 months) [5]. The MOH diagnosis was given during an in-person interview at the screening visit by an investigator who received specific training regarding the diagnosis of MOH. Preventive treatment of migraine (prescription or over-the-counter medication recommended by a healthcare professional) was allowed provided the dose and regimen was stable for ≥ 12 weeks prior to the screening visit and expected to be maintained until the end of treatment visit (Week 24).

In this study, a migraine day was defined as any day with a headache that meets the CM definition as outlined in the International Headache Society guidelines (section 1.3.1.1) [26] for controlled trials of preventive treatment of CM in adults. This includes any day with a headache longer than 4 h in duration, headache meeting ICHD-3 items C and D (migraine without aura), or a headache at least 30 min long plus aura symptoms. A migraine day was also defined based on patient perception of migraine severity; that is, a day with a headache at least 30 min long believed by the patient to be a migraine and for which the patient took a triptan, ergotamine, or other migraine-specific acute medication also met the criteria.

Adults were ineligible for study participation if previous anti-CGPR treatment(s) failed or if they had confounding and clinically significant pain syndromes, an acute or active temporomandibular disorder diagnosis, other headache type diagnosis, clinically significant cardiovascular disease, or an uncontrolled/untreated psychiatric condition for ≥ 6 months prior. Full inclusion and exclusion criteria are detailed in the protocol.

### Randomization

Patients were randomly allocated via an interactive response technology system to one of the two treatment groups: eptinezumab 100 mg or placebo, in a 1:1 ratio. Additionally, all patients were to receive eptinezumab 100 mg in the open-label period. Thus, no patient was denied access to active treatment with eptinezumab. The term “treatment sequence” is used to denote the treatment groups arising by combining the treatment received in the placebo-controlled period and the eptinezumab 100 mg received in the open-label period. Therefore, in the open-label period, the two treatment sequence groups were: placebo–eptinezumab 100 mg and eptinezumab 100 mg–eptinezumab 100 mg. The interactive response technology allocated patients to a treatment group and assigned a randomization number that was used to identify the patient throughout the study. Study site and number of MHDs (< 20/ ≥ 20 MHDs at baseline) data collected during the screening period was used to stratify the randomization.

### Study outcomes

The primary endpoint for efficacy was the change from baseline in MMDs over the 12-week placebo-controlled period (Weeks 1–12). Key secondary endpoints, listed in the testing hierarchy order, were the change from baseline in MMDs with use of AHM (Weeks 1–12), proportion of patients with ≥ 50% reduction from baseline in MMDs (migraine responder rate [MRR]; Weeks 1–12), migraine rate on the day after dosing (Day 1), proportion of patients with ≥ 75% reduction from baseline in MMDs (Weeks 1–4), change from baseline in the number of MHDs (Weeks 1–12), and proportion of patients with ≥ 75% reduction from baseline in MMDs (Weeks 1–12). Additional prespecified secondary and exploratory endpoints and the safety endpoints are summarized in Supplemental Table 1.

### Patient-reported outcomes

All patient-reported outcomes were administered in the local language and validated in the language to which they were translated. The Patient Global Impression of Change (PGIC) instructs patients to rate their improvement due to treatment and uses a rating system with 7 categories of change (“very much improved”, “much improved”, “minimally improved”, “no change”, “minimally worse”, “much worse”, and “very much worse”). The lower the score, the greater the patient’s perceived improvement in their disease-related functioning [27].

During the screening visit, investigators verbally asked patients for their patient-identified most bothersome symptom (PI-MBS) related to migraine, which was then categorized by the investigator into one of the following choices: nausea, vomiting, light sensitivity, sound sensitivity, mental cloudiness, fatigue pain with activity, mood changes, and “other/specify” (for alternative answers). Improvements were rated on a 7-point scale similar to that of PGIC, with lower scores indicating greater improvement in the most bothersome symptom [28]. Additional methods for patient-reported outcomes can be found in the Supplemental Methods section.

### Statistical analysis

In a prior study of eptinezumab, the subgroup of CM patients with MOH showed an improvement of 3.0 MMDs for the 100-mg dose compared to placebo, with a standard deviation of 6.0 [8]. Assuming the same effect size, 86 patients per treatment group provided a power of 90% for the primary endpoint using a 5% significance level. To account for 5% of randomized patients not contributing to the primary endpoint, 91 patients randomized per treatment group—or 182 randomized patients in total—provided a power of 90% to detect an effect size as mentioned for the MOH subgroup.

The estimand for the primary endpoint was described by the following attributes. The first attribute was the population of interest, which was patients with a dual diagnosis of migraine and MOH who fulfilled the inclusion and exclusion criteria of the study. The second attribute was the endpoint to be considered, which was the change from baseline in MMDs (Weeks 1–12). The third attribute was the treatment condition of interest, which was the comparison of eptinezumab 100 mg to placebo, with or without the use of preventive migraine medication. The fourth attribute was the other intercurrent event of interest, which was handled with a treatment policy strategy to assess the effect regardless of infusion interruption or termination before full dose is received. The last attribute was the population level summary, which was the mean difference in the primary endpoint across Weeks 1–12 comparing the effect of eptinezumab 100 mg to placebo.

The main estimator for the primary estimand was based on the primary endpoint, change from baseline in the number of MMDs (Weeks 1–12), which was estimated using a restricted maximum likelihood–based mixed model for repeated measures (MMRM) approach. The analysis was performed on MMDs by month using an MMRM, with month defined as 4-week intervals (Weeks 1–4, Weeks 5–8, Weeks 9–12), with baseline MMDs as a continuous covariate, and treatment, stratum (< 20 MHDs, ≥ 20 MHDs at baseline), month, and region as fixed factors. In addition, the model included treatment-by-month interaction, baseline MMDs-by-month interaction, and stratum-by-month interaction. Within-patient errors were modeled using an unstructured variance.

For the key secondary endpoints based on responder rates, treatment effects compared to placebo were analyzed using a logistic regression model that included MMDs at baseline as a continuous covariate, and treatment and stratification factor (< 20 MHDs, ≥ 20 MHDs at baseline) as factors. Migraine rate on the day after dosing (Day 1), was analyzed using a Cochran–Mantel–Haenszel test controlling for stratification factor (< 20 MHDs, ≥ 20 MHDs at baseline). Change from baseline in the number of MMDs with use of AHM (Weeks 1–12) and change from baseline in the number of MHDs (Weeks 1–12) were analyzed similarly to the primary endpoint.

The formal statistical testing of the primary endpoint and the 6 key secondary endpoints was done hierarchically, in a sequence of a maximum number of 7 steps. For each step, the treatment effect was tested on a 5% significance level, using a two-sided test, and testing only continued to the next step if all prior effects in the hierarchy were found to have p-values below the specified significance level (Supplemental Figure 2). For subgroup analyses, the analysis specified for the primary endpoint was repeated by region (Asia and Europe), sex, age group (≤ 35 years and > 35 years), the stratification factor (< 20 MHDs and ≥ 20 MHDs at baseline), and the number of previous preventive treatment failures (0, ≥ 1). Furthermore, a post hoc analysis of the primary and key secondary endpoints was presented separately for Chinese patients (i.e., patients from Mainland China and Taiwan).

## Results

### Study population

**Fig. 1:**
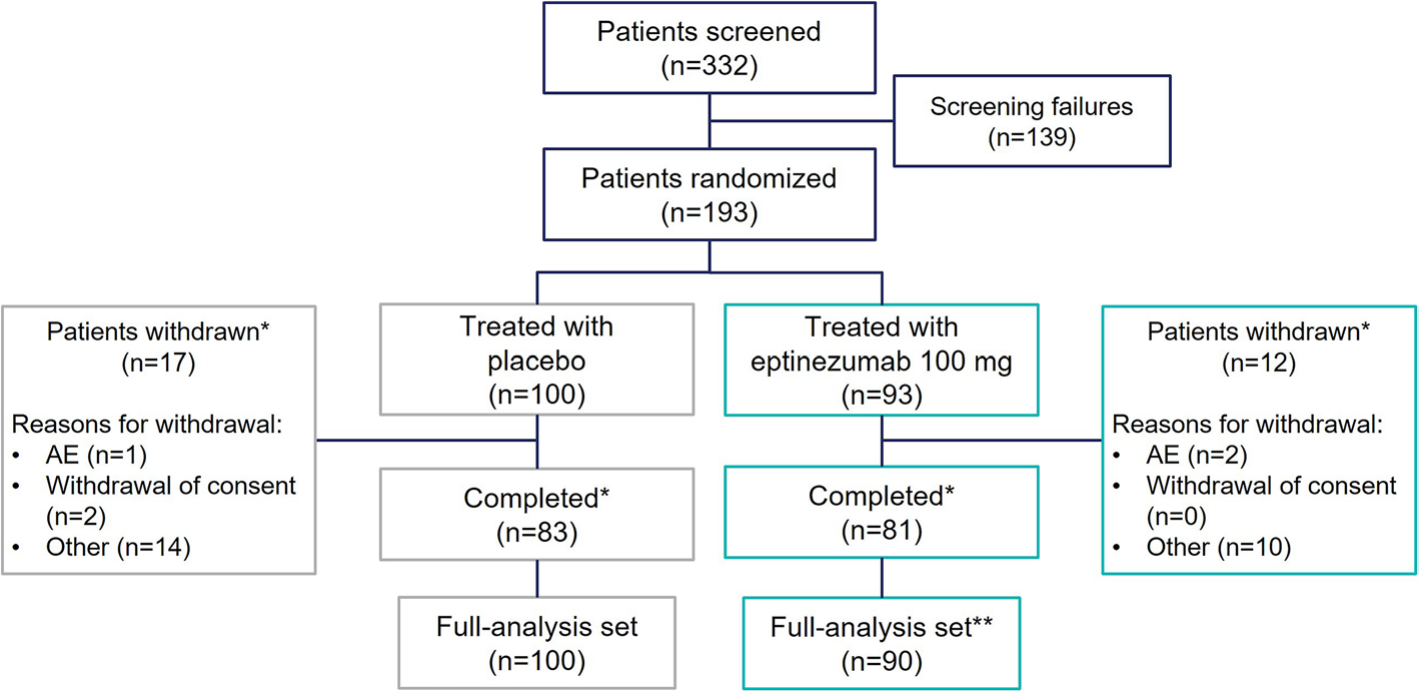
Patient disposition (placebo-controlled period). *Completed and withdrawn data refer to the number of patients completing or withdrawing in the placebo-controlled period. **Three patients from the eptinezumab group were excluded from the full analysis set because no post-baseline primary endpoint data were contributed. AE, adverse event

Between February 2021 and February 2022, a total of 332 patients were screened; 193 patients (the all-patients-treated set) with a dual diagnosis of migraine and MOH were randomized to eptinezumab 100 mg (*n* = 93) or placebo (*n* = 100). A total of 164 patients completed the placebo-controlled period (Fig. 1), and 29 patients withdrew. Baseline demographics and clinical characteristics of the full analysis set (FAS; *n* = 190; eptinezumab 100 mg [*n* = 90] and placebo [*n* = 100]) were generally similar between treatment groups. Three patients from the eptinezumab group were excluded from the FAS because no post-baseline primary endpoint data were contributed. Regarding the two treatment groups, most patients were female (148/190 [77.9%]), with a median age of 43.5 years. There was a slightly higher percentage of males in the eptinezumab group than in the placebo group (24/90 [26.7%] vs 18/100 [18%]; Table 1). Patients had on average 19.6 MMDs and 20.8 MHDs at baseline, with an average of 19.1 days per month of AHM use. The pattern of previous preventive treatment failures was similar across treatment groups; 57.4% of enrolled patients did not report previous preventive treatment failures (Supplemental Figure 3).

**Table 1:** Baseline demographics and clinical characteristics (FAS)

	**Placebo** **(*n* = 100)**	**Eptinezumab 100 mg** **(*n* = 90)**	**Total** **(*N* = 190)**
Sex, n (%)			
Male	18 (18.0)	24 (26.7)	42 (22.1)
Female	82 (82.0)	66 (73.3)	148 (77.9)
Age, median (interquartile range)	43.5 (35–52)	43.5 (37–54)	43.5 (35–52)
Region, n (%)			
Asia	81 (81.0)	74 (82.2)	155 (81.6)
Europe	19 (19.0)	16 (17.8)	35 (18.4)
Baseline characteristics, days (SD)			
Mean MMDs	19.7 (3.8)	19.5 (3.6)	19.6 (3.7)
Mean MHDs	20.9 (3.3)	20.6 (2.9)	20.8 (3.2)
Mean AHM use (days)	18.9 (4.5)	19.2 (4.6)	19.1 (4.6)
Baseline mean MMDs with use of AHM	19.2 (4.0)	18.9 (3.8)	19.1 (3.9)
Previous preventive treatment failures, n (%)			
Amitriptyline	15 (15.0)	12 (13.3)	27 (14.2)
Botulinum toxin A	13 (13.0)	10 (11.1)	23 (12.1)
Candesartan	2 (2.0)	0 (0.0)	2 (1.1)
Divalproex	1 (1.0)	1 (1.1)	2 (1.1)
Flunarizine	20 (20.0)	15 (16.7)	35 (18.4)
Metoprolol	4 (4.0)	3 (3.3)	7 (3.7)
Propranolol	10 (10.0)	9 (10.0)	19 (10.0)
Topiramate	20 (20.0)	16 (17.8)	36 (18.9)
Valproate	3 (3.0)	3 (3.3)	6 (3.2)
Other	24 (24.0)	18 (20.0)	42 (22.1)

*AHM* acute headache medication, *FAS* full analysis set, *MHD* monthly headache day, *MMD* monthly migraine day, *SD* standard deviation

### Efficacy outcomes

**Fig. 2:**
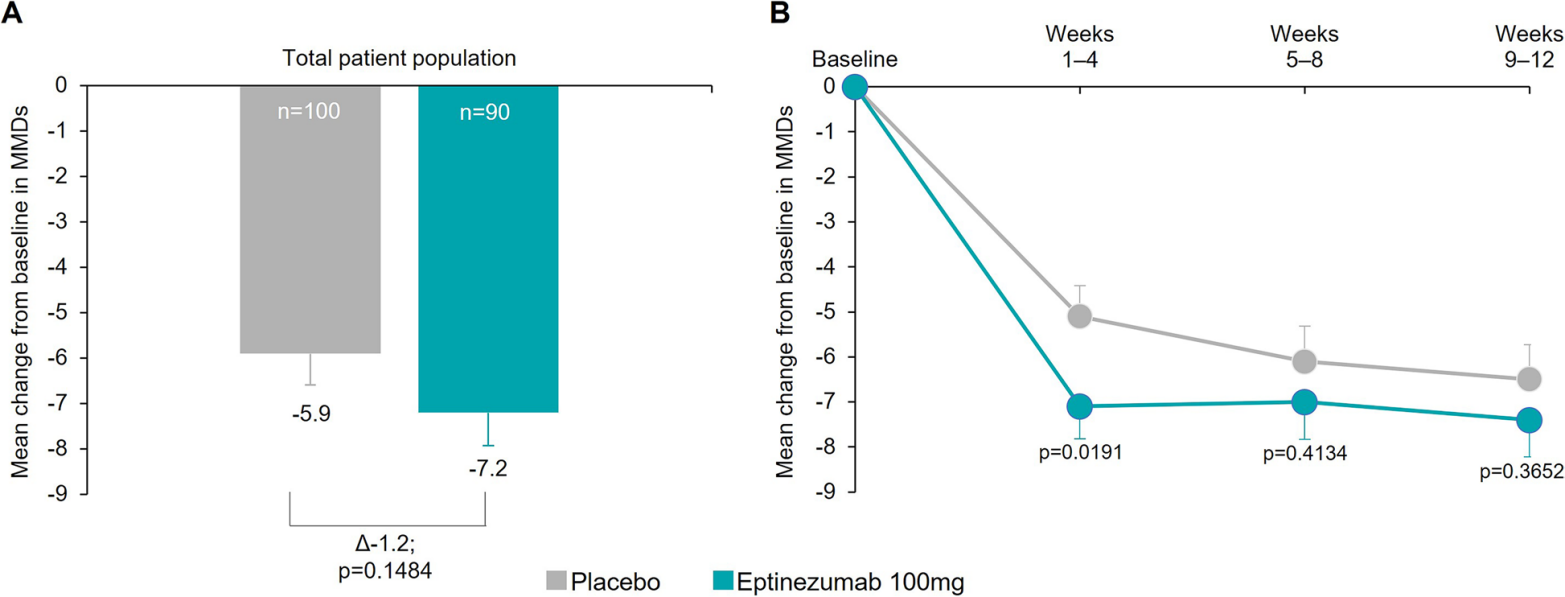
Change from baseline in mean MMDs (**A**) Weeks 1–12 and (**B**) 4-week intervals (FAS). The estimated means, mean differences from placebo, and 95% confidence intervals are from a mixed model for repeated measures with month (Weeks 1–4, Weeks 5–8, Weeks 9–12), region, stratification factor (monthly headache days at baseline: < 20/ ≥ 20), and treatment as factors, baseline score as a continuous covariate, treatment-by-month interaction, baseline score-by-month interaction, and stratum-by-month interaction. Data represent mean ± standard error. FAS, full analysis set; MMDs, monthly migraine days

At baseline, mean MMDs were similar across treatment groups (eptinezumab, 19.5; placebo, 19.7). Over Weeks 1–12, eptinezumab reduced mean MMDs more than placebo (difference from placebo [95% confidence interval] between treatments was -1.2 [-2.9 to 0.4]; *p* = 0.1484); i.e., this finding was not statistically significant (primary endpoint, Fig. 2a, Table 2). The reduction in MMDs over Weeks 1–4 showed greater reductions with eptinezumab (7.1 MMDs) than with placebo (5.1 MMDs; *p* = 0.0191 vs placebo; Fig. 2b). At baseline, the number of mean MMDs with AHM use was similar across treatment groups (eptinezumab, 18.9; placebo, 19.2). Changes from baseline in MMDs with AHM use over Weeks 1–12 followed a similar pattern, where eptinezumab reduced mean MMDs with AHM more than placebo (difference between treatments was -1.3; *p* = 0.1363; key secondary endpoint, Supplemental Figure 4a, Table 2). The reduction in MMDs with AHM use for patients treated with eptinezumab was greater over Weeks 1–4, with a reduction of 7.4 MMDs and 5.4 MMDs in the eptinezumab and placebo groups, respectively (*p* = 0.0196; Supplemental Figure 4b).

**Table 2:** Primary and key secondary efficacy outcomes (FAS)

	**Placebo** **(*n* = 100)**	**Eptinezumab 100 mg (*n* = 90)**
***Primary endpoint***		
Change from baseline in MMDs (Weeks 1–12; FAS)		
Change in mean from baseline (SE)	-5.9 (0.68)	-7.2 (0.73)
Difference from placebo (95% CI)		-1.2 (-2.9 to 0.4)
*p*-value vs placebo		0.1484
***Key secondary endpoints***		
Changes from baseline in MMDs with AHM (Weeks 1–12)		
Change in mean from baseline (SE)	-6.2 (0.69)	-7.5 (0.73)
Difference from placebo (95% CI)		-1.3 (-3.0 to 0.4)
*p*-value vs placebo		0.1363
≥ 50% reduction from baseline in MMDs (Weeks 1–12), n/N (%)	24/100 (24.0)	28/90 (31.1)
Difference to placebo (%)		7.1
Odds ratio vs placebo (95% CI)		1.45 (0.76 to 2.77)
*p*-value vs placebo		0.2563
Migraine rate on the day after dosing (Day 1)		
Baseline, n (%)	100 (70.5)	90 (69.5)
Day 1, n (%)	99 (59.2)	90 (44.2)
*p*-value vs placebo		0.0315
≥ 75% reduction from baseline in MMDs (Weeks 1–4), n/N (%)	1/99 (1.0)	16/90 (17.8)
Difference to placebo (%)		16.8
Odds ratio vs placebo (95% CI)		20.74 (4.07 to 378.98)
*p*-value vs placebo		< 0.0001
Change from baseline in the number of MHDs (Weeks 1–12)		
Change in mean from baseline (SE)	-5.9 (0.67)	-7.1 (0.70)
Difference from placebo (95% CI)		-1.2 (-2.9 to 0.5)
*p*-value vs placebo		0.1516
≥ 75% reduction from baseline in MMDs (Weeks 1–12), n/N (%)	2/100 (2.0)	15/90 (16.7)
Difference to placebo (%)		14.7
Odds ratio vs placebo (95% CI)		9.78 (2.64 to 63.44)
*p*-value vs placebo		0.0002

*AHM* acute headache medication, *CI* confidence interval, *FAS* full analysis set, *MHDs* monthly headache days, *MMDs* monthly migraine days, *SE* standard error

**Fig. 3:**
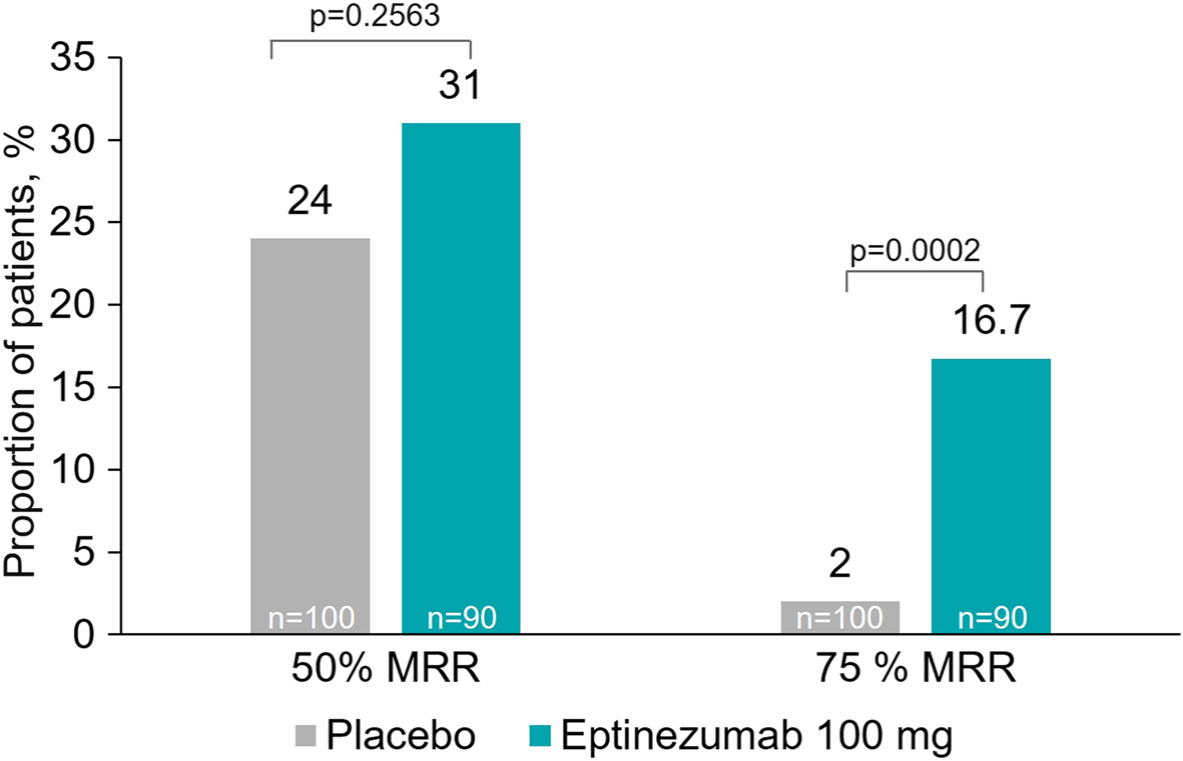
Patients with ≥ 50% and ≥ 75% reduction from baseline in MMDs over Weeks 1–12 (FAS). The 50% and 75% response variables across the three 4-week intervals are calculated as the average percentage change in MMDs (based on the available monthly values of MMDs). The comparison is based on logistic regression model including baseline MMDs as a continuous covariate, and treatment and stratification factor (monthly headache days at baseline: < 20/ ≥ 20) as factors. If the MMD value is missing for a given month, the responder status is derived based on the available values. n indicates the number of patients with observations. Data represent mean percentages. FAS, full analysis set; MMD, monthly migraine days; MRR, migraine responder rate

The eptinezumab group showed a numerically higher proportion of patients than the placebo group with ≥ 50% reductions from baseline in MMDs (31.1% compared to 24.0%, respectively; *p* = 0.2563; key secondary endpoint; Fig. 3, Table 2). Moreover, patients treated with eptinezumab during Weeks 1–12 were more likely than those treated with placebo to achieve, relative to baseline, a ≥ 75% in MMDs (16.7% compared to 2%, respectively; *p* = 0.0002; key secondary endpoint; Fig. 3, Table 2). A smaller percentage of patients treated with eptinezumab had migraine on the day after dosing compared to the placebo group (eptinezumab, 44.2%; placebo, 59.2%; *p* = 0.0315; Supplemental Figure 5, Table 2).

### Patient-reported outcomes

**Fig. 4:**
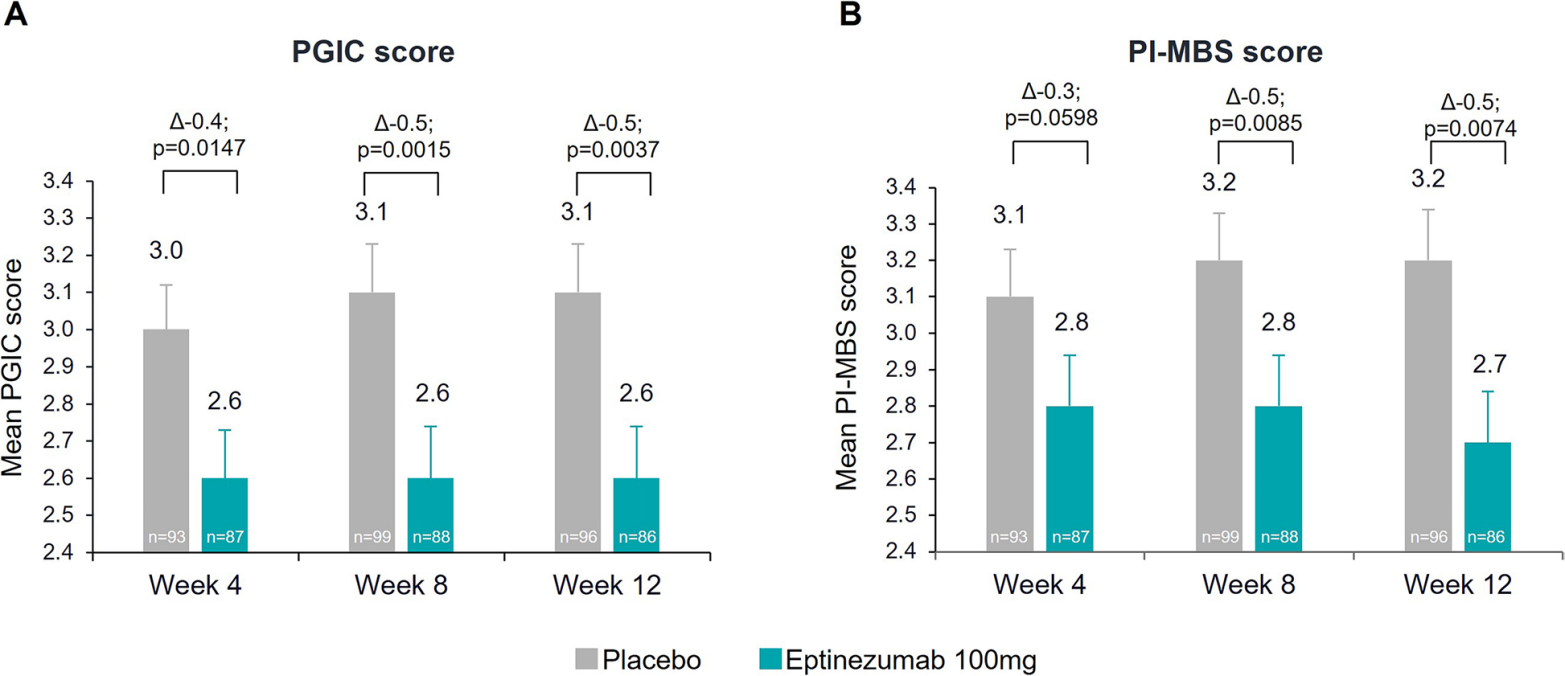
Patient Global Impression of Change (**A**) and patient-identified most bothersome symptom (**B**) scores (FAS). The model includes the following fixed effects: visit, region, stratification factor (monthly headache days at baseline: < 20/ ≥ 20), and treatment as factors, treatment-by-visit interaction, and stratum-by-visit interaction. The PGIC and the PI-MBS are ranked on a scale of 1–7, and the lower the score the higher the clinical improvement. Patients could rate their change on the PGIC and PI-MBS scale as “Very much improved”, “much improved”, “minimally improved”, “no change”, “minimally worse”, “much worse”, or “very much worse”. Data represent mean ± standard error. FAS, full analysis set; PGIC, Patient Global Impression of Change; PI-MBS, patient-identified most bothersome symptom

Larger improvements were observed in both PGIC and PI-MBS scores at Week 12 in the eptinezumab-treated group, with the mean PGIC scores being 2.6 for eptinezumab and 3.1 for placebo (*p* = 0.0037; Fig. 4) and the mean PI-MBS scores being 2.7 for eptinezumab and 3.2 for placebo (*p* = 0.0074). There was a higher proportion of patients achieving clinical significance (a 5-point reduction) in the 6-item Headache Impact Test (HIT-6) total score at Week 12 in the eptinezumab-treated group (57.6 eptinezumab vs 46.8 placebo; *p* = 0.0516; Supplemental Figure 6a) [29].

When analyzing the change from baseline in Migraine-Specific Quality of Life Questionnaire subscores at Week 12 between treatments, role function for both restrictive (*p* = 0.0445) and preventive (*p* = 0.0434) categories favored eptinezumab treatment (Supplemental Figure 6b). Overall improvement in EQ-5D-5L Visual Analog Scale (VAS) scores was greater in the eptinezumab-treated group over Weeks 1–12 (Supplemental Figure 7). Similarly, change from baseline in migraine Work Productivity and Activity Impairment (WPAI:M) subscores (absenteeism, presenteeism, work productivity loss, and activity impairment) were generally numerically in favor of the eptinezumab-treated patients (Supplemental Figure 8a-d) [30].

### Subgroup analyses: efficacy outcomes

A post hoc analysis in Chinese patients (*n* = 137), changes from baseline in MMDs over Weeks 1–12 followed a similar pattern as observed in the FAS, where eptinezumab reduced mean MMDs by 7.1 compared to 5.6 in the placebo group (*p* = 0.1584; Table 3). In the smaller subgroup of European patients (*n* = 35) in this study, the change from baseline in MMDs over Weeks 1–12 was more pronounced in the eptinezumab-treated group compared to the placebo group (8.6 mean MMD reduction compared to 5.4 in the placebo group [*p* = 0.1756; Table 4]), whereas in the complementary group (Asian patients, *n* = 155) the mean MMD reduction was 6.5 for the eptinezumab-treated group compared to 5.6 in the placebo group (*p* = 0.3280; Supplemental Table 2).

**Table 3:** Post hoc analyses of the primary and key secondary efficacy outcomes in Chinese patients

	**Placebo** **(*n* = 72)**	**Eptinezumab 100 mg (*n* = 65)**
***Primary endpoint***		
Change from baseline in MMDs (Weeks 1–12)		
Change in mean from baseline (SE)	-5.6 (0.71)	-7.1 (0.75)
Difference from placebo (95% CI)		-1.4 (-3.5 to 0.6)
*p*-value vs placebo		0.1584
***Key secondary endpoints***		
Changes from baseline in MMDs with AHM (Weeks 1–12)		
Change in mean from baseline (SE)	-5.8 (0.72)	-7.4 (0.76)
Difference from placebo (95% CI)		-1.6 (-3.7 to 0.4)
*p*-value vs placebo		0.1216
≥ 50% reduction from baseline in MMDs (Weeks 1–12), n/N (%)	16/72 (22.2)	21/65 (32.3)
Difference to placebo (%)		10.1
Odds ratio vs placebo (95% CI)		1.75 (0.81 to 3.87)
*p*-value vs placebo		0.1563
Migraine rate on the day after dosing (Day 1)		
Baseline, n (%)	72 (69.0)	65 (70.9)
Day 1, n (%)	71 (57.1)	65 (42.0)
*p*-value vs placebo		0.0612
≥ 75% reduction from baseline in MMDs (Weeks 1–4), n/N (%)	1/71 (1.4)	13/65 (20.0)
Difference to placebo (%)		18.6
Odds ratio vs placebo (95% CI)		20.12 (3.68 to 378.87)
*p*-value vs placebo		< .0001
Change from baseline in the number of MHDs (Weeks 1–12)		
Change in mean from baseline (SE)	-5.6 (0.70)	-7.0 (0.74)
Difference from placebo (95% CI)		-1.5 (-3.5 to 0.6)
*p*-value vs placebo		0.1548
≥ 75% reduction from baseline in MMDs (Weeks 1–12), n/N (%)	1/72 (1.4)	12/65 (18.5)
Difference to placebo (%)		17.1
Odds ratio vs placebo (95% CI)		19.04 (3.44 to 359.04)
*p*-value vs placebo		0.0001

The Chinese patient subpopulation was composed of patients from both Mainland China and Taiwan

*AHM* acute headache medication, *CI* confidence interval, *MHD* monthly headache days, *MMD* monthly migraine days, *SE* standard error

**Table 4:** Primary efficacy outcomes of subgroup analysis in European patients

	**Placebo** **(*n* = 19)**	**Eptinezumab 100 mg (*n* = 16)**
***Primary endpoint***		
Change from baseline in MMDs (Weeks 1–12)		
Change in mean from baseline (SE)	-5.3 (1.48)	-8.6 (1.73)
Difference from placebo (95% CI)		-3.3 (-8.0 to 1.5)
*p*-value vs placebo		0.1713

*CI* confidence interval, *MMDs* monthly migraine days, *SE* standard error

In women treated with eptinezumab (*n* = 148), a numerically greater difference from placebo in change from baseline in MMDs was observed than what was observed in men (*n* = 42; -1.6 [*p* = 0.0964] compared to -0.1 [*p* = 0.9662], respectively; Supplemental Table 2). Moreover, in patients with fewer previous preventive treatment failures, a post hoc analysis showed there was a numerically greater change from baseline for the eptinezumab-treated group when compared to placebo, with a difference of -1.6 MMDs for 0 previous preventive treatment failures (*p* = 0.1672) and -0.4 MMDs for ≥ 1 previous preventive treatment failure (*p* = 0.7424; Supplemental Table 2).

### Safety and tolerability

During the placebo-controlled period, vital signs, laboratory values, and electrocardiograms (ECGs) did not show any clinically relevant safety findings. Thirty-four percent of patients in the placebo group and 41% of patients in the eptinezumab group experienced treatment-emergent adverse events (TEAEs; Table 5). No TEAEs led to infusion interruption or termination. One TEAE in the placebo group and 2 in the eptinezumab group led to patient withdrawal from the study. Two serious adverse events were reported in the eptinezumab treatment group (1 acute myocardial infarction [the patient was withdrawn from the study and fully recovered] and 1 rib fracture).

**Table 5:** Placebo-controlled period summary of treatment-emergent adverse events (APTS)

	**Placebo** **(*n* = 100)**	**Eptinezumab 100 mg (*n* = 93)**
Patients with TEAEs, n (%)	34 (34.0)	38 (40.9)
Total number of TEAEs	59	74
TEAEs occurring in ≥ 2% of patients, n (%)	15 (15.0)	18 (19.4)
Upper respiratory tract infection	2 (2.0)	3 (3.2)
Dermatitis atopic	0 (0)	2 (2.2)
Diarrhea	1 (1.0)	2 (2.2)
Dizziness	2 (2.0)	2 (2.2)
Glycosylated haemoglobin increased	1 (1.0)	2 (2.2)
Muscle spasms	0 (0.0)	2 (2.2)
Nasopharyngitis	0 (0.0)	2 (2.2)
Nausea	2 (2.0)	2 (2.2)
Proteinuria	0 (0.0)	2 (2.2)
Urinary tract infection	1 (1.0)	2 (2.2)
Influenza-like illness	2 (2.0)	1 (1.1)
Fatigue	4 (4.0)	0 (0.0)
Urinary tract infection, bacterial	4 (4.0)	0 (0.0)
Patients with SAEs, n (%)	0 (0.0)	2 (2.2)
Total number of SAEs	0	2
Patients with TEAEs leading to infusion interruption/termination, n (%)	0 (0.0)	0 (0.0)
Total number of TEAEs leading to study drug infusion interruption/termination	0	0
Patients with TEAEs leading to withdrawal, n (%)	1 (1.0%)	2 (2.2%)
Total number of TEAEs leading to withdrawal	1	2
Deaths, n (%)	0 (0.0)	0 (0.0)

*APTS* all-patients-treated set, *SAE* serious adverse event, *TEAE* treatment-emergent adverse event

During the open-label period, vital signs, laboratory values, and ECGs did not reveal any clinically relevant safety findings. Forty-seven percent of patients randomized to the placebo − eptinezumab 100-mg treatment sequence group and 42% of patients randomized to the eptinezumab 100-mg − eptinezumab 100-mg treatment sequence group experienced TEAEs (Table 6). Similar to the placebo-controlled period, no TEAEs led to infusion interruption or termination. One TEAE in the placebo − eptinezumab 100-mg treatment sequence group and 1 in the eptinezumab 100-mg − eptinezumab 100-mg treatment sequence group led to patient withdrawal from the study. Seven serious adverse events were reported by 4 patients in the eptinezumab 100-mg − eptinezumab 100-mg treatment sequence group (preferred terms: bronchitis, headache, dermal cyst, intervertebral disc protrusion, radiculopathy, and pharyngitis).

**Table 6:** Open-label period summary of treatment-emergent adverse events presented by treatment sequence group (APTS-OL)

	**Placebo–eptinezumab 100 mg (*n* = 81)**	**Eptinezumab 100 mg–eptinezumab 100 mg** **(*n* = 81)**
Patients with TEAEs, n (%)	38 (46.9)	34 (42.0)
Total number of TEAEs	71	74
TEAEs occurring in ≥ 2% of patients, n (%)		
Migraine	1 (1.2)	5 (6.2)
Covid-19	3 (3.7)	3 (3.7)
Pyrexia	0 (0.0)	3 (3.7)
Urinary tract infection	0 (0.0)	3 (3.7)
Bronchitis	1 (1.2)	2 (2.5)
Glucose tolerance impaired	0 (0.0)	2 (2.5)
Headache	0 (0.0)	2 (2.5)
Myalgia	1 (1.2)	2 (2.5)
Pharyngotonsillitis	0 (0.0)	2 (2.5)
Dizziness	3 (3.7)	1 (1.2)
Abdominal pain upper	2 (2.5)	0 (0.0)
Diarrhea	2 (2.5)	0 (0.0)
Fatigue	2 (2.5)	0 (0.0)
Hyperlipidemia	3 (3.7)	0 (0.0)
Proteinuria	2 (2.5)	0 (0.0)
Upper respiratory tract infection	11 (13.6)	0 (0.0)
Patients with SAEs, n (%)	0 (0.0)	4 (4.9)
Total number of SAEs	0	7
Patients with TEAEs leading to infusion interruption/termination, n (%)	0 (0.0)	0 (0.0)
Total number of TEAEs leading to study drug infusion interruption/termination	0	0
Patients with TEAEs leading to withdrawal, n (%)	1 (1.2)	1 (1.2)
Total number of TEAEs leading to withdrawal	1	1
Deaths, n (%)	0 (0.0)	0 (0.0)

Due to 2 patients receiving erroneous study drug at visit 5, the APTS-OL consists of *n* = 162 patients when *n* = 164 patients completed the placebo-controlled period

*APTS-OL* all-patients-treated − open-label set, *SAE* serious adverse event, *TEAE* treatment-emergent adverse event

## Discussion

The objective of this study was to evaluate the efficacy and safety of eptinezumab to prevent migraine and headache in a predominantly Asian patient population with a dual diagnosis of migraine and MOH. Although the study’s primary endpoint did not meet statistical significance and the hierarchical testing strategy was stopped after the first primary endpoint hypothesis test, the data consistently trended in favor of eptinezumab treatment versus placebo, with p-values below 0.05 observed in 3 out of the 6 key secondary endpoints (migraine on the day after dosing, ≥ 75% reduction from baseline in MMDs [Weeks 1–4], ≥ 75% reduction from baseline in MMDs [Weeks 1–12], as well as in the PGIC and PI-MBS patient perception of change).

There can be many reasons why the primary endpoint of the study was not met. One reason is a smaller effect size than anticipated, and another reason is that the sample size was smaller than previous studies of eptinezumab in migraine prevention studies [19, 21, 31]. Moreover, there might be both extrinsic and intrinsic ethnic factors involved, causing this trial population to be potentially different when compared to the global migraine population from previous trials. This could be because of cultural differences in healthcare and migraine management, including clinical research (such as differences in clinical trial recruitment between different countries or how patients report headache/migraine characteristics). Differences observed in this study may show that these two patient populations (European and Asian) may be interpreting headache and migraine definitions differently (e.g., considering non-migraine headaches to be migraine), leading to different results. Similarly, there may be a difference in patient reporting between the primary endpoint and secondary endpoints. All these aspects constitute examples of potential extrinsic ethnic factors that might play a role here. The fact that the headache diary does not tell the same story as the patient-reported outcomes may in fact show that the different cultures interact differently with this eDiary and with the clinical practice in clinical trials. The estimated change in MMDs for European patients were similar to the effect seen in previous eptinezumab trials [19, 21, 31]. This study contained a higher percentage of men; therefore, sex might constitute an intrinsic factor, making the study potentially less comparable to the general migraine population from previous trials.

Patients with MOH were included in this study and there are differences between Asian and European patients, including the overused medications and clinical manifestations of MOH, which can also partly explain the results [32]. To meet MOH diagnostic criteria according to ICHD-3 criteria, patients must have a primary headache disorder with headache on ≥ 15 days per month in conjunction with overuse of acute treatments (defined as ≥ 10 or ≥ 15 days per month depending on the medication class) [5]. At baseline, mean MMDs with use of AHM (19.1 days) in the SUNLIGHT population suggests that these participants had had room for improvement in both MMD and AHM use which may have contributed to a larger than expected placebo effect. In the placebo group and the eptinezumab group, improvements in changes from baseline in MMDs with AHM over Weeks 1–12 were observed (-6.2 and -7.5, respectively). In patients with CM and MOH, reduction of AHM use may be an effective strategy to decrease MMDs and headache severity [32]. Similar observations have been observed in other studies with participants with MOH [33, 34]. Despite the present study not including patient education or behavioural interventions in a standardized way, patients may have spontaneously reduced their use of AHM knowing of their inclusion in an MOH-study. This may have been a contributing factor to improvement in both treatment groups and the lack of significance observed for the primary endpoint. Other aspects that may have contributed include the fact that AHM use/reduction was not controlled or that combination analgesics with unknown ingredients may have difficult-to-predict efficacy and washout times.

Of note, in the SUNLIGHT patient population, 57% of the enrolled patients had no previous preventive treatment failures. In the previous subgroup analysis of patients with CM and MOH in a larger study from the US and Europe, all patients treated with eptinezumab reported prior use of an oral preventive [22]. This lack of previous preventive treatment failures for headache/migraine may have led to a higher placebo response rate as well as the cultural factors previously discussed [21]. Importantly, like previously published studies [19, 21, 31], eptinezumab was well tolerated in the patient population studied here. In both the placebo-controlled and open-label periods, no new safety signals were identified.

### Limitations

This was a comparatively small study population in which the statistical power was set using a large, assumed difference in the reduction of MMDs from baseline to Weeks 1–12 (sample size assumptions were based on the MOH subgroup from PROMISE-2) [31]. Moreover, patients with previous anti-CGRP therapy failures, as well as clinically significant cardiovascular disease or confounding pain syndromes, were excluded from participation; therefore, the findings may not be indicative of safety and efficacy in patients with these or other excluded conditions.

## Conclusions

This study did not meet its primary efficacy endpoint; it is therefore negative. Overall, however, all efficacy endpoints numerically favored eptinezumab treatment when compared to placebo. In addition, the SUNLIGHT study had similar safety and tolerability compared to previous trials in the overall study population and in the Asian patients, who represented the majority of the population. Moreover, the patient-reported outcome results were aligned with expectations of eptinezumab treatment increasing quality of life. Like previous studies, eptinezumab was proven to be well tolerated in both the placebo-controlled and the open-label period of this study.

### Supplementary Information

**Additional file 1:** **Supplemental Table 1.** Study objectives and endpoints. **Supplemental Table 2.** Analysis of change from baseline in MMDs (Weeks 1–12) across various subgroups (FAS). **Supplemental Figure 1.** SUNLIGHT study design. **Supplemental Figure 2.** Statistical testing hierarchy for primary and key secondary endpoints. **Supplemental Figure 3.** Number of previous preventive treatment failures (FAS). **Supplemental Figure 4.** Change from baseline in MMDs with AHM use over (A) Weeks 1–12 and (B) 4-week intervals (FAS). **Supplemental Figure 5.** Percentage of patients with migraine on the day after the first dose (FAS). **Supplemental Figure 6.** Analysis of (A) proportion of patients achieving a 5-point reduction in HIT-6 total score at Week 12 and (B) change from baseline in MSQ subscores at Week 12 (FAS). **Supplemental Figure 7.** Mean change from baseline in EQ-5D-5L VAS score (FAS). **Supplemental Figure 8.** Change from baseline in WPAI:M subscores: (A) absenteeism, (B) presenteeism, (C) work productivity loss, and (D) activity impairment (FAS).

## Data Availability

In accordance with The European Federation of Pharmaceutical Industries and Associations (EFPIA) and the Pharmaceutical Research and Manufacturers of America (PhRMA) Principles for Responsible Clinical Trial Data Sharing guidelines, H. Lundbeck is committed to responsible sharing of clinical trial data in a manner that is consistent with safeguarding the privacy of patients, respecting the integrity of national regulatory systems, and protecting the intellectual property of the sponsor. The protection of intellectual property ensures continued research and innovation in the pharmaceutical industry. Deidentified data are available to those whose request has been reviewed and approved through an application submitted to https://www.lundbeck.com/global/our-science/clinical-data-sharing.

## Abbreviations

AHM: Acute headache medication
APTS: All-patients-treated set
APTS-OL: All-patients-treated-open-label set
CGRP: Calcitonin gene-related peptide
CM: Chronic migraine
ECG: Electrocardiogram
ICHD-3: International Classification of Headache Disorders, 3rd edition
IV: Intravenous
FAS: Full analysis set
HIT-6: 6-Item Headache Impact Test
MOH: Medication-overuse headache
MMDs: Monthly migraine days
MHDs: Monthly headache days
MMRM: Mixed model for repeated measures
MMR: Migraine responder rate
MSQ: Migraine-Specific Quality of Life Questionnaire
PGIC: Patient Global Impression of Change
PI-MBS: Patient-identified most bothersome symptom
TEAEs: Treatment-emergent adverse events
WPAI:M: Work Productivity and Activity Impairment.
VAS: Visual Analog Scale
