# Supplementary material for: Efficacy and safety of eptinezumab in patients with chronic migraine and medication-overuse headache: a randomized, double-blind, placebo-controlled study
Source: BMC Neurol. 2023 Dec 15;23:441. doi: 10.1186/s12883-023-03477-z (PMC10722704; doi:10.1186/s12883-023-03477-z)
Supplement: Supplementary file 1 — Additional file 1: Supplemental Table 1. Study objectives and endpoints. Supplemental Table 2. Analysis of change from baseline in MMDs (Weeks 1–12) across various subgroups (FAS). Supplemental Figure 1. SUNLIGHT study design. Supplemental Figure 2. Statistical testing hierarchy for primary and key secondary endpoints. Supplemental Figure 3. Number of previous preventive treatment failures (FAS). Supplemental Figure 4. Change from baseline in MMDs with AHM use over (A) Weeks 1–12 and (B) 4-week intervals (FAS). Supplemental Figure 5. Percentage of patients with migraine on the day after the first dose (FAS). Supplemental Figure 6. Analysis of (A) proportion of patients achieving a 5-point reduction in HIT-6 total score at Week 12 and (B) change from baseline in MSQ subscores at Week 12 (FAS). Supplemental Figure 7. Mean change from baseline in EQ-5D-5L VAS score (FAS). Supplemental Figure 8. Change from baseline in WPAI:M subscores: (A) absenteeism, (B) presenteeism, (C) work productivity loss, and (D) activity impairment (FAS). [file 12883_2023_3477_MOESM1_ESM.pdf]

Supplemental Materials

Supplemental Methods

Patient-reported outcomes

Supplemental Tables and Figures

Supplemental Table 1. Study objectives and endpoints

Supplemental Table 2. Analysis of change from baseline in MMDs (Weeks 1–12) across various subgroups (FAS)

Supplemental Figure 1. SUNLIGHT study design

Supplemental Figure 2. Statistical testing hierarchy for primary and key secondary endpoints

Supplemental Figure 3. Number of previous preventive treatment failures (FAS)

Supplemental Figure 4. Change from baseline in MMDs with AHM use over (A) Weeks 1–12 and (B) 4-week intervals (FAS)

Supplemental Figure 5. Percentage of patients with migraine on the day after the first dose (FAS)

Supplemental Figure 6. Analysis of (A) proportion of patients achieving a 5-point reduction in HIT-6 total score at Week 12 and (B) change from baseline in MSQ subscores at Week 12 (FAS)

Supplemental Figure 7. Mean change from baseline in EQ-5D-5L VAS score (FAS)

Supplemental Figure 8. Change from baseline in WPAI:M subscores: (A) absenteeism, (B) presenteeism, (C) work productivity loss, and (D) activity impairment (FAS)

Supplemental Methods

Patient-reported outcomes

6-item Headache Impact Test (HIT-6): HIT-6 (v1.0) provides patients with a way to describe the impacts of headache on normal daily function. The brief questionnaire consists of six scored questions, each with the following choices for response: "never" (6 points), "rarely" (8 points), "sometimes" (10 points), "very often" (11 points), and "always" (13 points). The total score is calculated by adding the points associated with each response, providing a total score that ranges from 36 to 78. From these scores, the severity of headache effect on daily life is determined [1].

Migraine-Specific Quality of Life Questionnaire (MSQ): MSQ (v2.1) assesses the impact of migraine on patient quality of life based on three categories of assessment: role function restrictive, role function preventive, and emotional function. For each of the fourteen total items spanning the three categories, eac h item is scored using a scale from 1 to 6, and subsequent raw scores are transformed to a scale ranging from 0 to 100, with higher scores indicating better patient quality of life [2-4].

EQ-5D-5L Visual Analogue Scale (VAS): The EQ-5D-5L assesses patient well-being using the descriptive categories of mobility, self-care, usual activities, pain/discomfort, and depression/anxiety along with a visual analogue scale (VAS). The brief assessment describes well-being using a scale ranging from the worst imaginable health state (score 0) to the best imaginable health state (score 100) [5].

Work Productivity and Activity Impairment: Migraine (WPAI:M): Patients use the WPAI:M assessment to obtain a measurement of their work productivity and activity impairment in the context of their migraine-related health problems. The six-item questionnaire explores the impact of migraine on life such as the number of hours worked, the numbers of working hours missed, effects on work productivity, and effects on normal daily activities outside of work. Lower scores indicate a better quality of life compared to higher scores [6].

References

1. Kosinski M, Bayliss MS, Bjorner JB, Ware JE, Garber WH, Cady R, et al. A six-item short-form survey for measuring headache impact: the HIT-6. Qual Life Res. 2003; 12(8): 963–974.
2. Jhingran P, Osterhaus JT, Miller DW, Lee JT, Kirchdoerfer L. Development and validation of the Migraine-Specific Quality of Life Questionnaire. Headache. 1998; 38(4): 295–302.
3. Cole JC, Lin P, Rupnow MFT. Validation of the Migraine-Specific Quality of Life Questionnaire version 2.1 (MSQ v. 2.1) for patients undergoing prophylactic migraine treatment. Qual Life Res. 2007;16(7):1231-1237.
4. Rendas-Baum R, Bloudek LM, Maglinte GA, Varon SF. The psychometric properties of the Migraine-Specific Quality of Life Questionnaire version 2.1 (MSQ) in chronic migraine patients. Qual Life Res. 2013;22(5):1123-1133.
5. Herdman M, Gudex C, Lloyd A, Janssen M, Kind P, Parkin D, et al. Development and preliminary testing of the new five-level version of EQ-5D (EQ-5D-5L). Quality of Life Research. 2011; 20: 1727–1736.
6. Reilly MC, Zbrozek AS, Dukes, EM. The validity and reproducibility of a work productivity and activity impairment instrument. Pharmacoeconomics. 1993; 4(5): 353–365.

Supplemental Tables and Figures

Supplemental Table 1. Study objectives and endpoints

Objectives	Endpoints
**Primary Objective**<br>To evaluate the efficacy of eptinezumab for the prevention of migraine and medication-overuse headache (MOH)	**Primary endpoint**<br>- Change from baseline in the number of monthly migraine days (MMDs) (Weeks 1–12)<br>**Key secondary endpoints**<br>- Change from baseline in MMDs with use of acute medication (Weeks 1–12)<br>- Response: ≥50% reduction from baseline in MMDs (Weeks 1–12)<br>- Migraine on the day after dosing (Day 1)<br>- Response: ≥75% reduction from baseline in MMDs (Weeks 1–4)<br>- Change from baseline in the number of monthly headache days (MHDs) (Weeks 1–12)<br>- Response: ≥75% reduction from baseline in MMDs (Weeks 1–12)<br><br>**Secondary endpoints**<br>- Response: ≥75% reduction from baseline in MHDs (Weeks 1–12)<br>- Response: ≥75% reduction from baseline in MHDs (Weeks 1–4)<br>- Change from baseline in the number of MHDs with use of acute medication (Weeks 1–12)<br>- Change from baseline in the proportion of migraine attacks with severe pain intensity (Weeks 1–12)<br>- Change from baseline in the proportion of headache episodes with severe pain intensity (Weeks 1–12)<br>- Patient Global Impression of Change (PGIC) score at Week 12<br>- Change score at Week 12 in patient-identified most bothersome symptom (PI-MBS; as reported at Screening)<br><br>**Exploratory endpoints**<br>- Response: 100% reduction from baseline in MMDs (average of 4-weeks results over Weeks 1–12)<br>- Response: 100% reduction from baseline in MHDs (average of 4-weeks results over Weeks 1–12)<br>- Change from baseline in monthly number of migraine attacks (Weeks 1–12)<br>- Change from baseline in monthly number of headache episodes (Weeks 1–12)<br>- Change from baseline in monthly days with use of acute migraine medication (Weeks 1–12)<br>- Change from baseline in monthly days with use of ergotamine (Weeks 1–12, Weeks 1–4, Weeks 5–8, Weeks 9–12); similar endpoints defined for use of triptans, analgesics, opioids, combination analgesics, traditional Chinese medicines (TCMs), antiemetics, analginum, antipyrine, and tolfenamic acid<br>- Change from baseline in monthly days with use of nonopioid analgesics (defined as any of the following 4 categories of non-opioid analgesics collected in the eDiary: analgesic, analginum, antipyrine, and tolfenamic acid medications) (Weeks 1–12, Weeks 1–4, Weeks 5–8, Weeks 9–12)

	- Shifts in the use of medication from above/below the medication overuse (MO) threshold at baseline to above/below the MO threshold at Weeks 1–4, Weeks 5–8, and Week 9–12; the MO threshold for each class of drugs is presented below:<br>o Ergotamine ≥10 days/month<br>o Triptans ≥10 days/month<br>o Non-opioid analgesics ≥15 days/month<br>o Opioids ≥10 days/month<br>o Combination-analgesics ≥10 days/month<br>o Any combination of ergotamine, triptans, non-opioid analgesics, and/or opioids on ≥10 days/month
**Secondary Objective**<br>To evaluate the efficacy of eptinezumab on health-related quality of life, health care resource utilization, and work productivity	**Secondary endpoints**<br>- Change from baseline to Week 12 in the Headache Impact Test (HIT-6) score<br>- Response: ≥5-point reduction from Baseline to Week 12 in HIT-6 total score<br>- Change from baseline to Week 12 in the Migraine-Specific Quality of Life (MSQ v2.1) subscores<br>- Change from baseline to Week 12 in the health-related quality of life (EQ-5D-5L Visual Analogue Scale [VAS]) score<br>- Baseline and Week 12 in health care resources utilization (HCRU)<br>- Change from baseline to Week 12 in the Migraine Work Productivity and Activity Impairment Questionnaire (WPAI:M) subscores
**Exploratory Objective**<br>To obtain patient input on experience with migraine and MOH, treatment-meaningful change, and benefit-risk trade-off assessments	**Exploratory Endpoints**<br>- Patients' characteristics and experiences at Week 12 Visit in terms of:<br>o Impact on daily activities/health-related quality of life<br>o Impact on social and professional life<br>o Interpretation of quantitative assessments and endpoints to discuss meaningfulness of change<br>o Experience with past preventive treatments<br>o Experience with product regimen (e.g., frequency and mode of administration)<br>o Overall treatment satisfaction
**Safety Objective**<br>To evaluate the safety and tolerability of eptinezumab	**Safety Endpoints**<br>- Adverse events<br>- Absolute values and changes from baseline in clinical safety laboratory test values, vital signs, weight, and electrocardiogram (ECG) parameter values<br>- Potentially clinically significant clinical safety laboratory test values, vital signs, weight changes, and ECG parameter values<br>- Development of specific anti-eptinezumab antibodies (ADAs), such as neutralizing antibodies (NAbs)<br>- Columbia Suicide Severity Rating Scale (CSSRS) score

Supplemental Table 2. Analysis of change from baseline in MMDs (Weeks 1–12) across various subgroups (FAS)

	**Placebo**	**Eptinezumab 100 mg**
Subgroup analyses of primary endpoint		
**Change from baseline in MMDs in males (Weeks 1–12)**		
Number of patients	18	24
Change in mean from baseline (SE)	-3.6 (2.61)	-3.7 (2.66)
Difference from placebo (95% CI)		-0.1 (-4.3 to 4.1)
p-value vs placebo		0.9662
**Change from baseline in MMDs in females (Weeks 1–12)**		
Number of patients	82	66
Change in mean from baseline (SE)	-5.8 (0.72)	-7.4 (0.78)
Difference from placebo (95% CI)		-1.6 (-3.5 to 0.3)
p-value vs placebo		0.0964
**Change from baseline in MMDs in Asian* patients (Weeks 1–12)**		
Number of patients	81	74
Change in mean from baseline (SE)	-5.6 (0.66)	-6.5 (0.69)
Difference from placebo (95% CI)		-0.9 (-2.8 to 0.9)
p-value vs placebo		0.3280
**Change from baseline in MMDs in European patients (Weeks 1–12)**		
Number of patients	19	16
Change in mean from baseline (SE)	-5.4 (1.49)	-8.6 (1.74)
Difference from placebo (95% CI)		-3.2 (-8.0 to 1.5)
p-value vs placebo		0.1756
**Change from baseline in MMDs in ≤35 years of age (Weeks 1–12)**		
Number of patients	27	21
Change in mean from baseline (SE)	-7.8 (1.31)	-9.2 (1.60)
Difference from placebo (95% CI)		-1.4 (-5.0 to 2.1)
p-value vs placebo		0.4202
**Change from baseline in MMDs in >35 years of age (Weeks 1–12)**		
Number of patients	73	69
Change in mean from baseline (SE)	-5.4 (0.78)	-6.5 (0.79)
Difference from placebo (95% CI)		-1.2 (-3.0 to 0.7)
p-value vs placebo		0.2188
**Change from baseline in MMDs in <20 MHDs (Weeks 1–12)**		
Number of patients	42	36
Change in mean from baseline (SE)	-5.4 (1.03)	-6.8 (1.07)
Difference from placebo (95% CI)		-1.5 (-3.8 to 0.9)
p-value vs placebo		0.2146
**Change from baseline in MMDs in ≥20 MHDs (Weeks 1–12)**		
Number of patients	58	54
Change in mean from baseline (SE)	-6.3 (0.91)	-7.2 (0.98)
Difference from placebo (95% CI)		-0.9 (-3.4 to 1.5)
p-value vs placebo		0. 4385

	**Placebo**	**Eptinezumab 100 mg**
Additional post hoc analyses		
**Change from baseline in MMDs in 0 preventive treatment failures (Weeks 1–12)**		
Number of patients	55	54
Change in mean from baseline (SE)	-6.9 (2.26)	-8.5 (2.25)
Difference from placebo (95% CI)		-1.6 (-4.0 to 0.7)
p-value vs placebo		0.1672
**Change from baseline in MMDs in ≥1 preventive treatment failures (Weeks 1–12)**		
Number of patients	45	36
Change in mean from baseline (SE)	-5.5 (0.86)	-5.9 (1.02)
Difference from placebo (95% CI)		-0.4 (-3.0 to 2.1)
p-value vs placebo		0.7424

*Note: The Asian subpopulation was composed of patients from Mainland China, Taiwan, and Republic of Korea. CI, confidence interval; FAS, full analysis set; MHD, monthly headache days; MMD, monthly migraine days; SE, standard error.

Supplemental Figure 1. SUNLIGHT study design

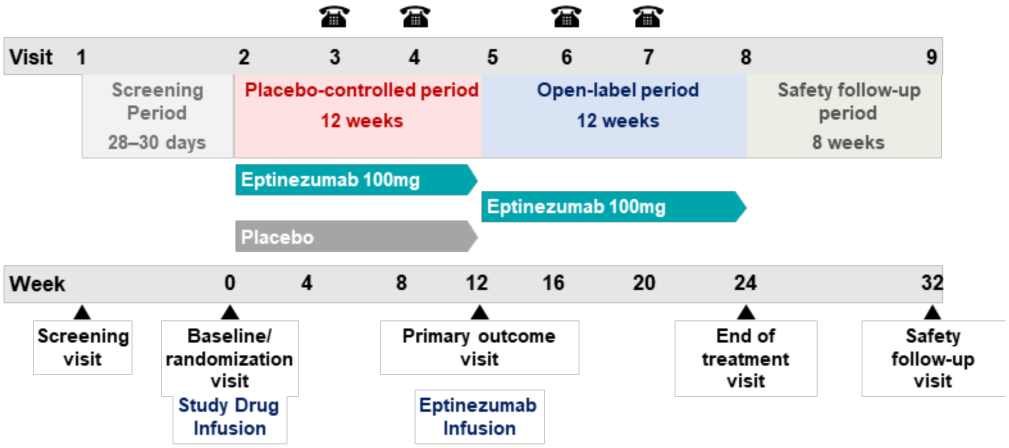

The study consisted of a screening period (28–30 days), a placebo-controlled period (12 weeks), an open-label period (12 weeks), and a safety follow-up period (8 weeks). Study drug (eptinezumab 100 mg or placebo) was administered by intravenous infusion at the baseline visit and at the primary outcome visit (Week 12). At Week 12, patients entered the 12-week open-label period. Patients returned to the clinic 8 weeks later for a safety follow-up visit. Study visits during the placebo-controlled period consisted of 2 phone contacts (Visit 3 and Visit 4) and a primary outcome visit (Visit 5). During the open-label period there were 2 phone contacts (Visit 6 and Visit 7) and an end-of-trial visit (Visit 8).

Supplemental Figure 2. Statistical testing hierarchy for primary and key secondary endpoints

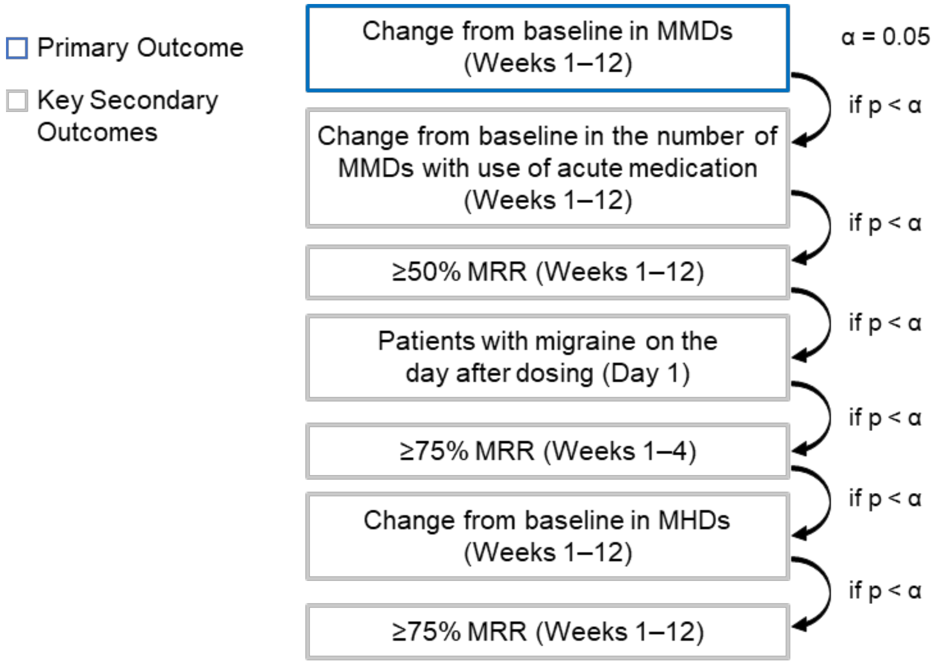

Statistical testing was done hierarchically, in a number of steps. For each step, the treatment effect was tested on a 5% significance level and testing only continued to the next step if all prior effects in the hierarchy were found to have p-values below the specified significance level.

MMDs, monthly migraine days; MHDs, monthly headache days; MRR, migraine responder rate.

Supplemental Figure 3. Number of previous preventive treatment failures (FAS)

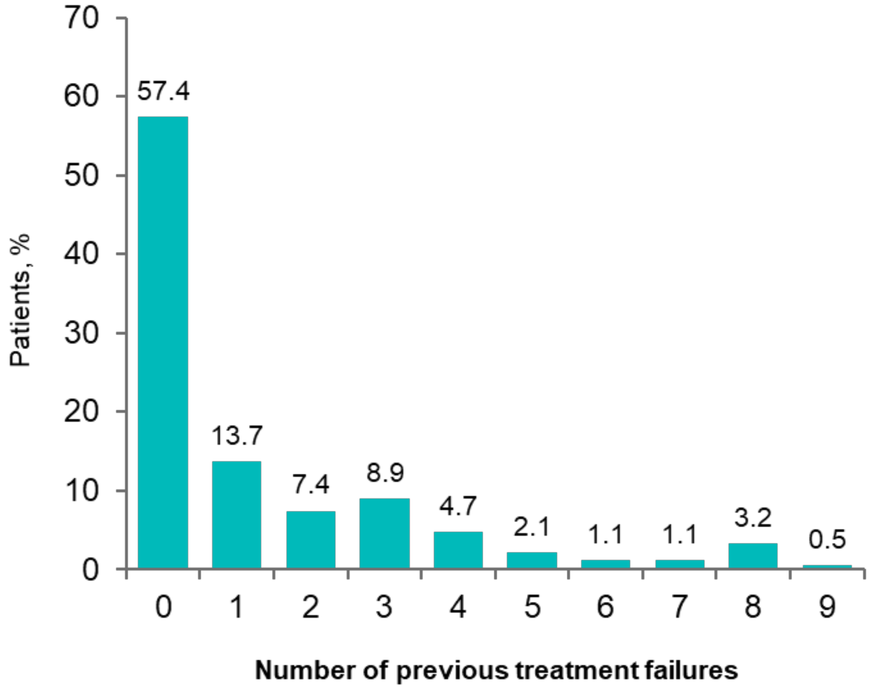

FAS, full analysis set.

Supplemental Figure 4. Change from baseline in MMDs with AHM use over (A) Weeks 1–12 and (B) 4-week intervals (FAS)

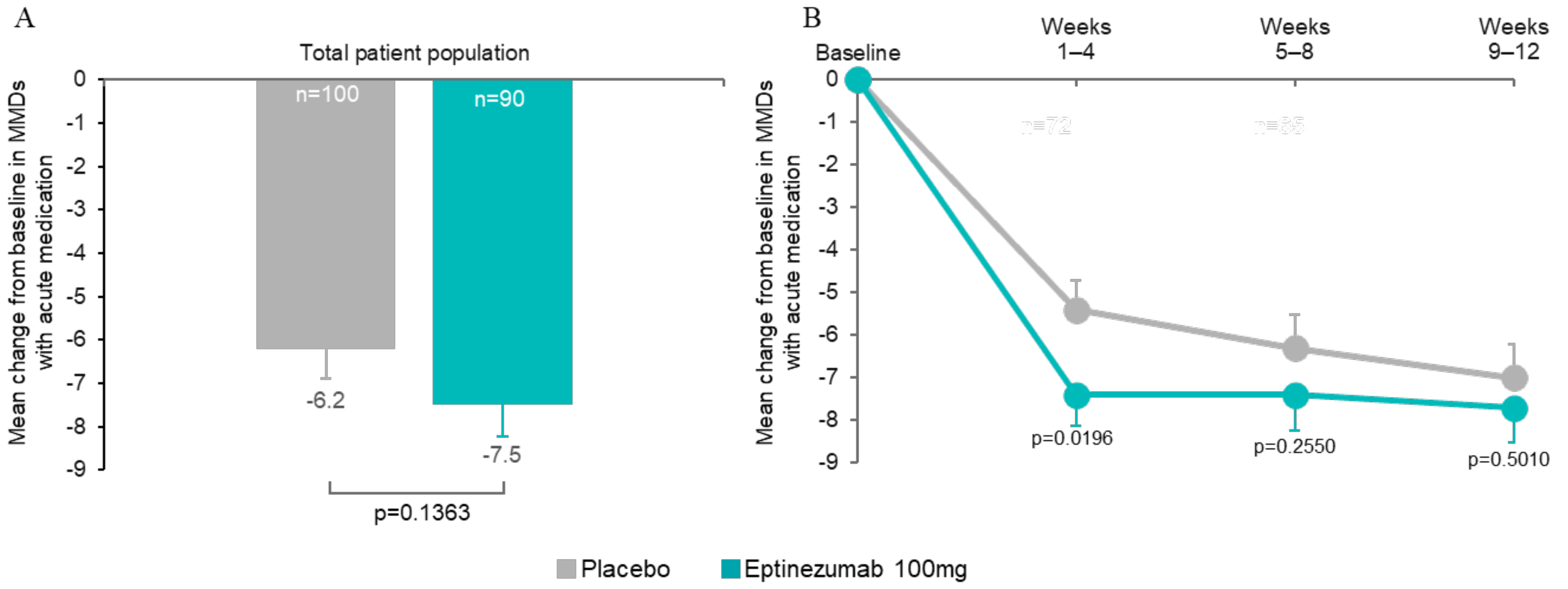

The estimated means, mean differences from placebo, and 95% confidence intervals are from an MMRM with month (Weeks 1–4, Weeks 5–8, Weeks 9–12), region, stratification factor (MHDs at baseline: <20/≥20) and treatment as factors, baseline score as a continuous covariate, treatment-by-month interaction, baseline score-by-month interaction, and stratum-by-month interaction. From the MMRM, estimates and tests across multiple 4-week intervals are computed via SAS using equal weights for each 4-week interval. Data represent mean ± standard error.

AHM, acute headache medication; FAS, full analysis set; MHDs, monthly headache days; MMDs, monthly migraine days; MMRM, mixed model for repeated measures; SAS, statistical analysis system.

Supplemental Figure 5. Percentage of patients with migraine on the day after the first dose (FAS)

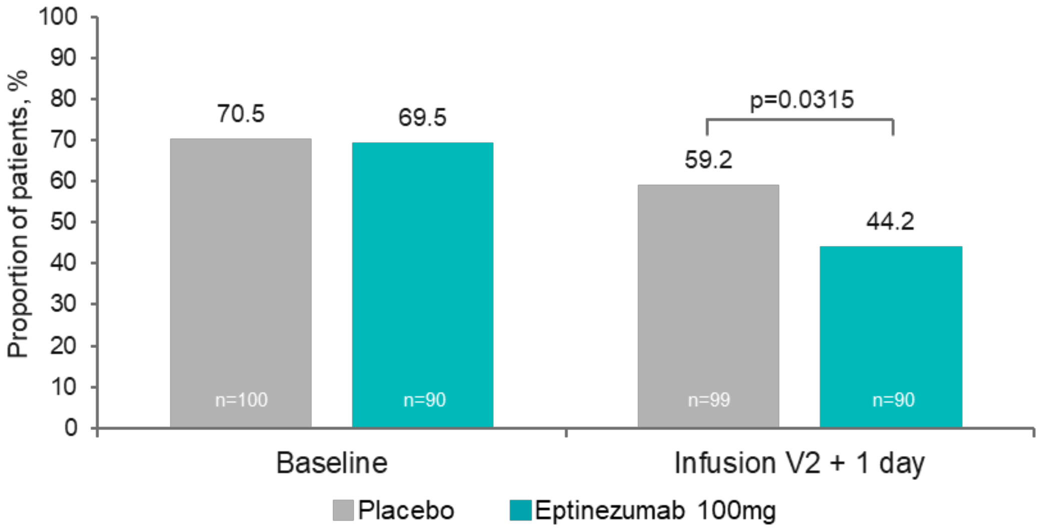

The p-values are computed using extended Cochran–Mantel–Haenszel test, adjusting for the stratification factor (monthly headache days at baseline <20/≥20). Baseline is the average percentage of patients with migraine across the first 28 days. The percentage of patients with a migraine on the day after first dosing is derived based on available eDiary data on Day 1, unless the eDiary data on Day 1 are missing. In that case, the migraine rate for the patient is imputed.

n indicates the number of subjects in the analysis at the relevant timepoint. FAS, full analysis set.

Supplemental Figure 6. Analysis of (A) proportion of patients achieving a 5-point reduction in HIT-6 total score at Week 12 and (B) change from baseline in MSQ subscores at Week 12 (FAS)

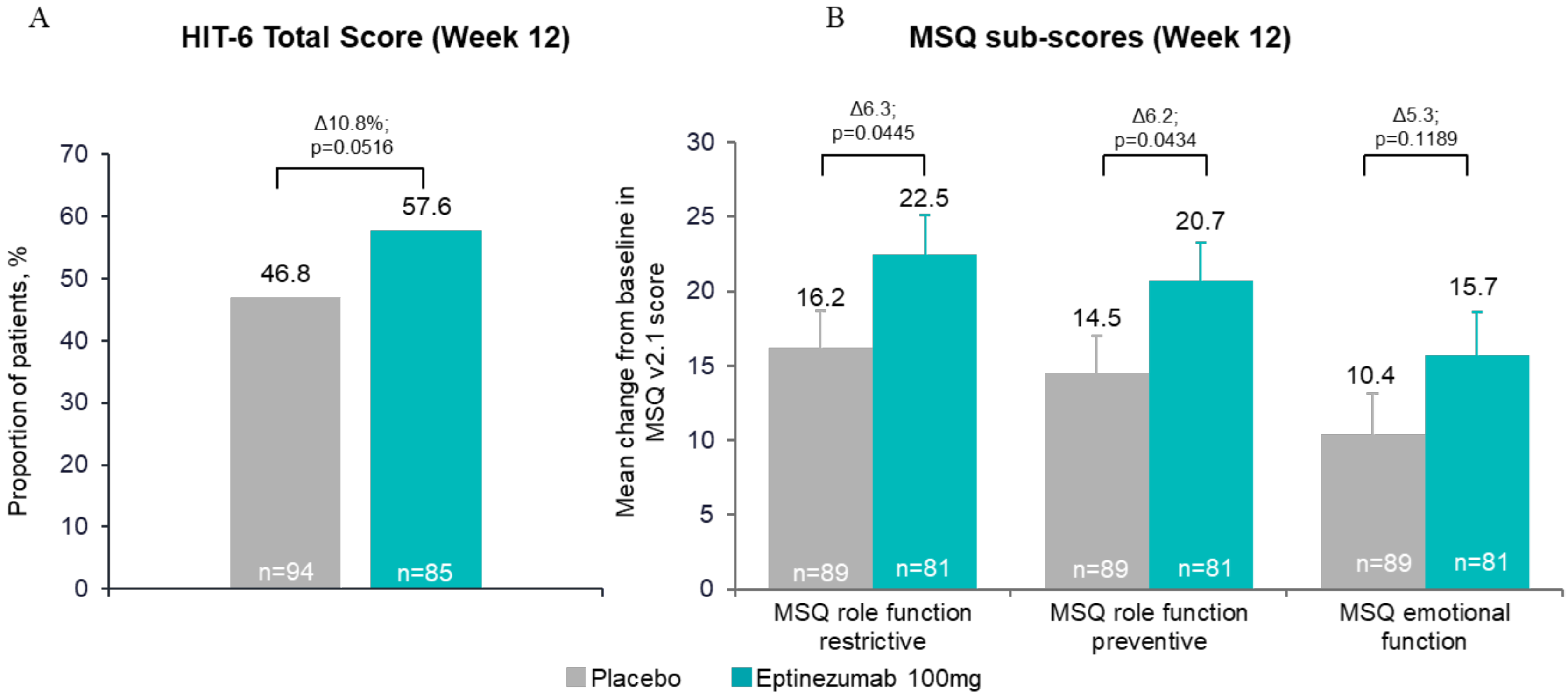

(A) Data represent mean ± standard error. n.s., not significant vs placebo. (B) The model includes the following fixed effects: visit, region, stratification factor (monthly headache days at baseline: <20/≥20), and treatment as factors, baseline MSQ v2.1 subscores as a continuous covariate, baseline score-by-visit interaction, treatment-by-visit interaction, and stratum-by-visit interaction. Data represent mean ± standard error.

FAS, full analysis set; HIT-6, 6-item Headache Impact Test; MSQ, Migraine-Specific Quality of Life Questionnaire, v2.1.

Yu, Zhou, Luo, et al. Efficacy and safety of eptinezumab in patients with chronic migraine and medication-overuse headache: a randomized, double-blind, placebo-controlled study. *BMC Neurology*. 2023.

Supplemental Figure 7. Mean change from baseline in EQ-5D-5L VAS score (FAS)

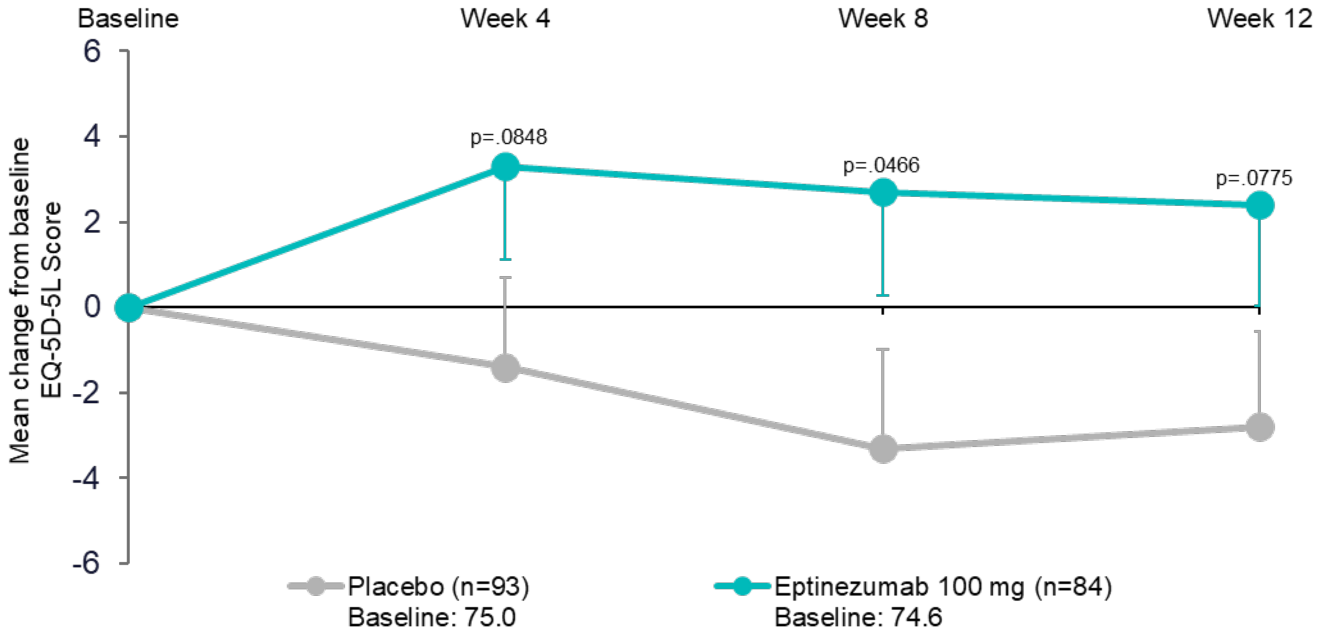

The model includes the following fixed effects: visit, region, stratification factor (monthly headache days at baseline: <20/≥20), and treatment as factors, baseline EQ-5D-5L VAS score as a continuous covariate, baseline score-by-visit interaction, treatment-by-visit interaction, and stratum-by-visit interaction. Data represent mean ± standard error.

FAS, full analysis set; VAS, visual analogue scale.

Supplemental Figure 8. Change from baseline in WPAI:M subscores: (A) absenteeism, (B) presenteeism, (C) work productivity loss, and (D) activity impairment (FAS)

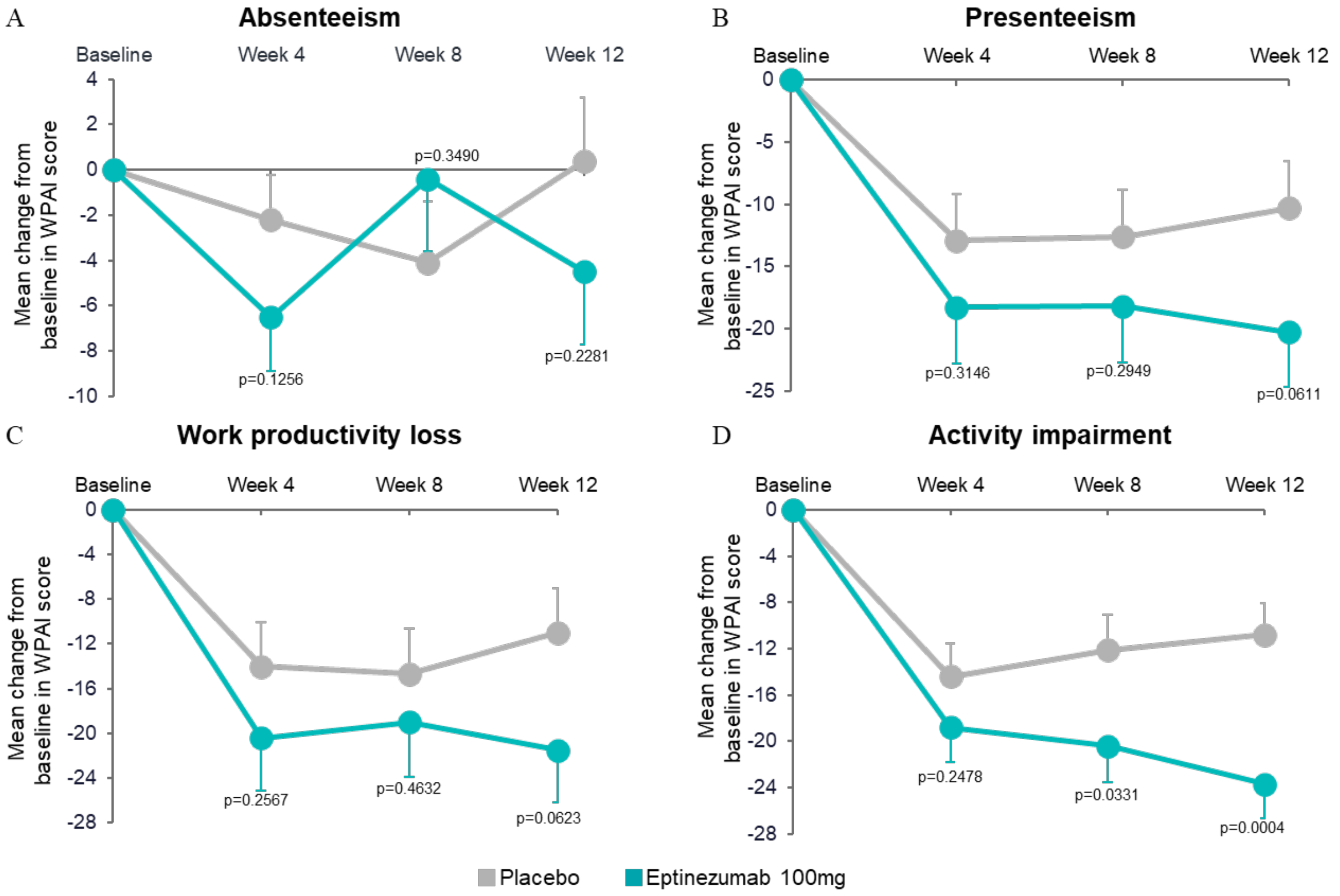

The model includes the following fixed effects: visit, region, stratification factor (MHDs at baseline: <20/≥20), and treatment as factors, baseline EQ-5D-5L VAS score as a continuous covariate, baseline score-by-visit interaction, treatment-by-visit interaction, and stratum-by-visit interaction. Data represent mean ± standard error. A decrease from baseline in WPAI:M score indicates improvement. FAS, full analysis set; MHDs, monthly headache days; VAS, visual analogue scale; WPAI:M, migraine work productivity and activity impairment.
